# Effect of Echinochrome A on Submandibular Gland Dysfunction in Ovariectomized Rats

**DOI:** 10.3390/md20120729

**Published:** 2022-11-22

**Authors:** Ji-Min Kim, Sung-Chan Shin, Yong-Il Cheon, Hyung-Sik Kim, Gi-Cheol Park, Hyoung-Kyu Kim, Jin Han, Jung-Eun Seol, Elena A. Vasileva, Natalia P. Mishchenko, Sergey A. Fedoreyev, Valentin A. Stonik, Byung-Joo Lee

**Affiliations:** 1 Medical Research Institute, College of Medicine, Pusan National University, Yangsan 50612, Republic of Korea; 2 Department of Otorhinolaryngology-Head and Neck Surgery, College of Medicine, Biomedical Research Institute, Pusan National University, Pusan National University Hospital, Busan 49241, Republic of Korea; 3 Department of Life Science in Dentistry, College of Dentistry, Pusan National University, Yangsan 50612, Republic of Korea; 4 Institute for Translational Dental Science, Pusan National University, Yangsan 50612, Republic of Korea; 5 Department of Otolaryngology-Head and Neck Surgery, Samsung Changwon Hospital, College of Medicine, Sungkyunkwan University, Changwon 51353, Republic of Korea; 6 Department of Physiology, College of Medicine, Cardiovascular and Metabolic Disease Center, Smart Marine Therapeutic Center, Department of Health Sciences and Technology, Graduate School, Inje University Busan, Busan 49241, Republic of Korea; 7 Department of Dermatology, Inje University Busan Paik Hospital, Inje University College of Medicine, Busan 49241, Republic of Korea; 8 G.B. Elyakov Pacific Institute of Bioorganic Chemistry, Far-Eastern Branch of the Russian Academy of Science, 690022 Vladivostok, Russia

**Keywords:** ovariectomy, menopause, xerostomia, submandibular gland, ferroptosis

## Abstract

Post-menopausal dry mouth or xerostomia is caused by reduced salivary secretion. This study aimed to investigate the efficacy of echinochrome A (Ech A) in alleviating submandibular gland dysfunctions in ovariectomized rats that mimic menopause. Female rats that were eight-weeks-old were randomly divided into SHAM-6, -12; OVX-6, -12; and ECH-6, -12 groups (consisting of 6- and 12-weeks post-sham-operated, ovariectomized, and Ech A-treated ovariectomized rats, respectively). The ECH groups had lower body weight than OVX but similar food intake and estradiol or estrogen receptor β expression. However, the ECH groups had lower mRNA expression of sterol-regulatory element binding protein-1c (*Srebp-1c*), acetyl-CoA carboxylase (*Acc*), fatty acid synthase (*Fasn*), cluster of differentiation 36 (*Cd36*), and lipid vacuole deposition than OVX mice. Moreover, reactive oxygen species (ROS), malondialdehyde (MDA), and iron accumulation were lower in the ECH than in the OVX groups. Fibrosis markers, transforming growth factor β (*Tgf-βI* and *Tgf-βII mRNA*) increased in the OVX than SHAM groups but decreased in the ECH groups. Aquaporin (*Aqp-1* and *Aqp-5 mRNA*) and mucin expressions were downregulated in the OVX groups but improved with Ech A. In addition, Ech A prevented post-menopausal salivary gland dysfunction by inhibiting lipogenesis and ferroptosis. These findings suggest Ech A as an effective remedy for treating menopausal dry mouth.

## 1. Introduction

Menopause is a physical condition that typically appears in women between the ages of 45–55 and is characterized by vasomotor symptoms, osteoporosis, genitourinary symptoms, and an elevated risk of cardiovascular diseases [1,2,3]. Eye and mouth dryness are prevalent during menopause, in addition to the usual physical symptoms [4]. Xerostomia is frequent in post-menopausal women and is linked to age and systemic illnesses [5,6]. Menopause-related changes in sex hormones can decrease salivary flow, causing hyposalivation, and xerostomia [7]. Xerostomia can severely affect the quality of life of post-menopausal women by causing speaking difficulties, dysphagia, dental caries, taste changes, halitosis, and burning mouth [8].

Menopause-related xerostomia and salivary gland dysfunctions are not well-characterized. Menopause induces obesity and tissue fat accumulation [9,10]. Kwon et al. reported that menopausal xerostomia might be linked to ferroptosis, enhanced lipogenesis, and lipid peroxidation in the salivary glands of ovariectomized rats [11]. Ferroptosis, a regulated cell death, is characterized by ROS generation, lipid peroxidation, glutathione peroxidase 4 (GPX4) depletion, lipid hydroperoxide accumulation, and iron availability [12,13]. However, no study has evaluated the impact of anti-ferroptotic agents on menopause-induced xerostomia. Antioxidants that alter the redox status are effective therapeutic agents for inhibiting ferroptosis [14,15]. Therefore, we hypothesized that suppressing ferroptosis by inhibiting ROS and lipid peroxidation using antioxidants would be an effective therapeutic strategy for menopause-induced dry mouth.

Echinochrome A (Ech A), found in different sea urchin species, including *Scaphechinus mirabilis*, has strong free-radical-scavenging properties [16,17]. Ech A is registered as a medicinal product and is approved for medicinal use in Russia (PubChem CID: 135457951, C12H10O7) [18,19]. Numerous studies over the last decade have demonstrated the safety and efficacy of Ech A in a wide range of diseases [20,21]. Ech A is anti-inflammatory, antimicrobial, and antioxidative [22,23,24]. In diabetic mice, Ech A administration increases the expression of antioxidants, such as glutathione-S-transferase, superoxide dismutase, and catalase [25,26,27]. In this study, we aimed to explore the mechanism by which Ech A affects post-menopausal submandibular gland function in ovariectomized rats that simulate menopause.

## 2. Results

### 2.1. Effect of Ech A on Basic Health Conditions

**Figure 1:**
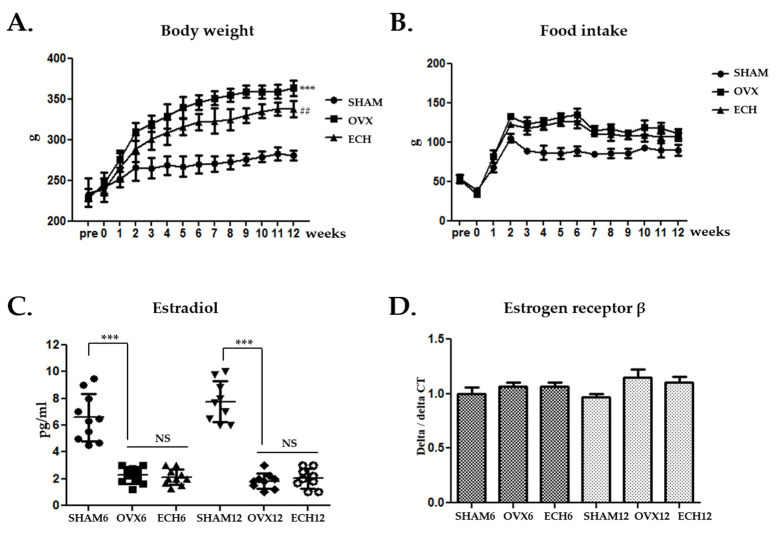
Effect of Ech A on body weight, food intake, and estradiol levels following an ovariectomy. (**A**) The body weight and (**B**) food intake increased in the OVX groups. Ech A treatment suppressed body weight, but there was no difference in food intake compared to the OVX group. (**C**) The serum estradiol levels were lower in the OVX groups and were not affected by Ech A treatment. (**D**) Quantitative polymerase chain reaction analysis of ErβII; estrogen receptor β did not differ between the groups. *n* = 8, each group. Error bar; S.D. One-way ANOVA; *** *p* < 0.001 vs. SHAM, ## *p* < 0.01; vs. OVX.

Ovariectomy-induced weight gain in the animals is primarily due to increased adipose tissue. After three to twelve weeks following an ovariectomy, the OVX groups gained significantly more weight and consumed more food than the SHAM groups (OVX-6 and 12 vs. SHAM-6 and 12, respectively; *p* < 0.001). When compared to the respective OVX groups, the Ech A treatment groups (ECH) had significantly lower body weights (ECH-6 vs. OVX-6; *p* < 0.05 and ECH-12 vs. OVX-12; *p* < 0.01) but no change in food intake (Figure 1A,B). Serum estradiol concentrations decreased in the OVX groups (OVX-6 and 12 vs. SHAM-6 and 12, respectively; *p* < 0.001). However, the estradiol concentrations that were treated with Ech A were not different from in the OVX rats, indicating that Ech A did not affect levels of estradiol (Figure 1C). Estrogen receptor (Erβ) mRNA in the submandibular glands was quantified using real-time polymerase chain reaction (PCR) for in vivo analysis of sex hormone receptor expression. No statistically significant differences were observed between the SHAM, OVX, and ECH groups (Figure 1D).

### 2.2. Ech A Suppressed Lipid Accumulation

**Figure 2:**
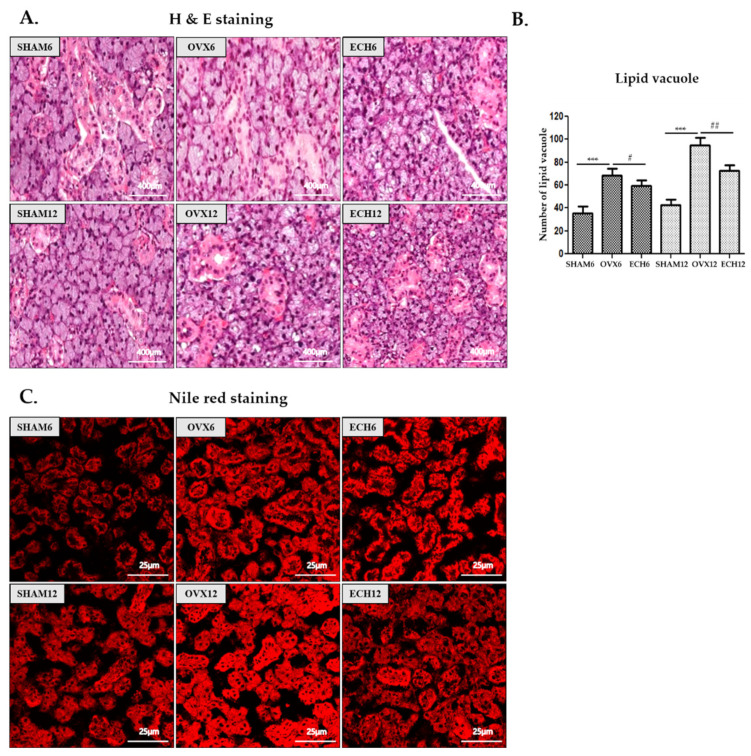
Effect of Ech A on lipid deposition. The lipid deposition was higher in the OVX groups but lower in the Ech A treatment groups. (**A**) Lipid vacuoles (yellow arrows) were detected in the submandibular gland after H and E staining. We counted intracellular lipid vacuoles, primarily distributed between acini cells or in the cytoplasm of serous cells. (**B**) Morphometric analysis of lipid vacuoles. (**C**) Nile red staining of rat submandibular gland. Positive Nile red staining was revealed in the cytoplasm of acinar cells, increased in the OVX groups, and decreased in the ECH groups of in the cytoplasm of acinar cells. *n* = 8, each group. Error bar; S.D. One-way ANOVA; *** *p* < 0.001; vs. SHAM, # *p* < 0.05, ## *p* < 0.01; vs. OVX.

Hematoxylin-eosin staining and Nile red staining was performed to detect general morphological alterations. An overall increase in the lipid vacuoles was detected in the submandibular gland tissues of the OVX groups. The data indicated that lipid accumulation was elevated in both the OVX groups (OVX-6 and 12 vs. SHAM-6 and 12, respectively; *p* < 0.001). However, these effects were significantly reduced in the Ech A treatment groups (ECH-6 vs. OVX-6; *p* < 0.05 and ECH-12 vs. OVX-12; *p* < 0.01) (Figure 2A,B). Following Nile red staining of rat submandibular gland, positive staining area was revealed in the cytoplasm of acinar cells, increased in OVX groups, and decreased in the ECH groups of in the cytoplasm of acinar cells.

### 2.3. Ech A Inhibited the Expression of Lipid Metabolic Genes

**Figure 3:**
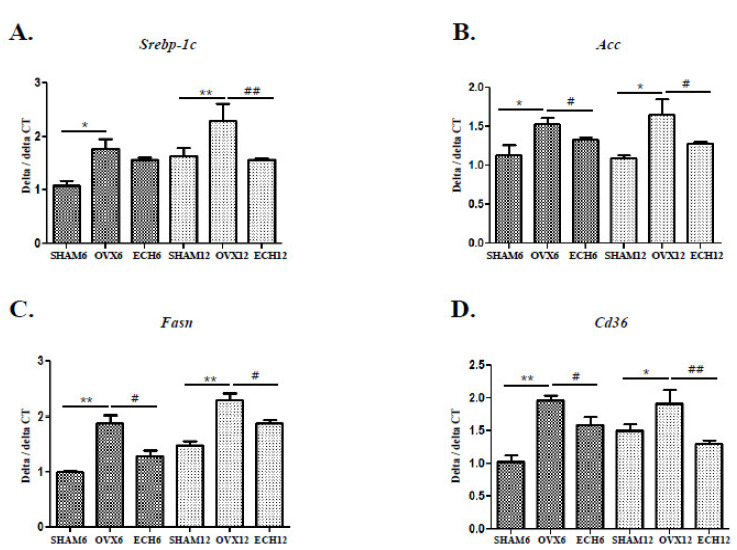
Effect of Ech A on lipid metabolic gene expression. The mRNA levels of lipid metabolic genes were higher in the SHAM groups but decreased in Ech A-treated groups. Quantitative polymerase chain reaction (qPCR) analysis of (**A**) Srebp-1c; sterol-regulatory element binding protein-1c. OVX groups (OVX-6 vs. SHAM-6; *p* < 0.05 and OVX-12 vs. SHAM-12; *p* < 0.01) had higher Srebp-1c mRNA levels but decreased in the ECH-12 group (*p* < 0.01 compared to OVX-12). (**B**) Acc; acetyl-CoA carboxylase. Acc mRNA expression was significantly higher in the OVX groups than in the SHAM groups (OVX-6 and 12 vs. SHAM-6 and 12, respectively; *p* < 0.05), but it was lower in the ECH groups (ECH-6 and 12 vs. OVX-6 and 12, respectively; *p* < 0.05). (**C**) Fasn; fatty acid synthase. Fasn mRNA expression was much higher in the OVX groups than in the SHAM groups (OVX-6 and 12 vs. SHAM-6 and 12, respectively; *p* < 0.01), but it was lower in the ECH groups (ECH-6 and 12 vs. OVX-6 and 12, respectively; *p* < 0.05). (**D**) Cd36; cluster of differentiation 36. Cd36 mRNA expression was much higher in the OVX groups than in the SHAM groups (OVX-6 vs. SHAM-6; *p* < 0.01 and OVX-12 vs. SHAM-12; *p* < 0.05), while it was much lower in the ECH groups (ECH-6 vs. OVX-6; *p* < 0.05 and ECH-12 vs. OVX-12; *p* < 0.01). *n* = 8, each group. Error bar; S.D. One-way ANOVA; * *p* < 0.05 and ** *p* < 0.01; vs. SHAM, # *p* < 0.05, ## *p* < 0.01; vs. OVX.

Several transcription factors and genes that are known to regulate lipid metabolism were investigated. The effects on sterol regulatory element-binding protein-1c (Srebp-1c) mRNA levels were initially explored. The Srebp-1c levels increased in the OVX groups but decreased significantly in the ECH-12 groups (Figure 3A). Subsequently, we examined whether Ech A treatment affected the expression of acetyl-CoA carboxylase (Acc) target genes (Figure 3B). The ACC is a lipid metabolic-related enzyme that catalyzes the irreversible carboxylation of acetyl-CoA to malonyl-CoA [28]. The Acc mRNA expression was significantly higher in the OVX groups but decreased following Ech A treatment. A key enzyme in the endogenous lipogenesis pathway, fatty acid synthase (FASN), is also a Srebp-1c target gene [29]. Fasn mRNA expression was significantly higher in the OVX groups but reduced in the ECH groups (Figure 3C). The cluster of differentiation 36 (Cd36) enhances fatty acid uptake and promotes inflammation [28,29]. Cd36 mRNA expression was significantly elevated in the OVX groups, but Ech A treatment decreased the effects (Figure 3D). These findings could explain the reduction in lipid vacuoles in salivary gland tissues following Ech A treatment.

### 2.4. Ech A Reduced Ferroptosis

**Figure 4:**
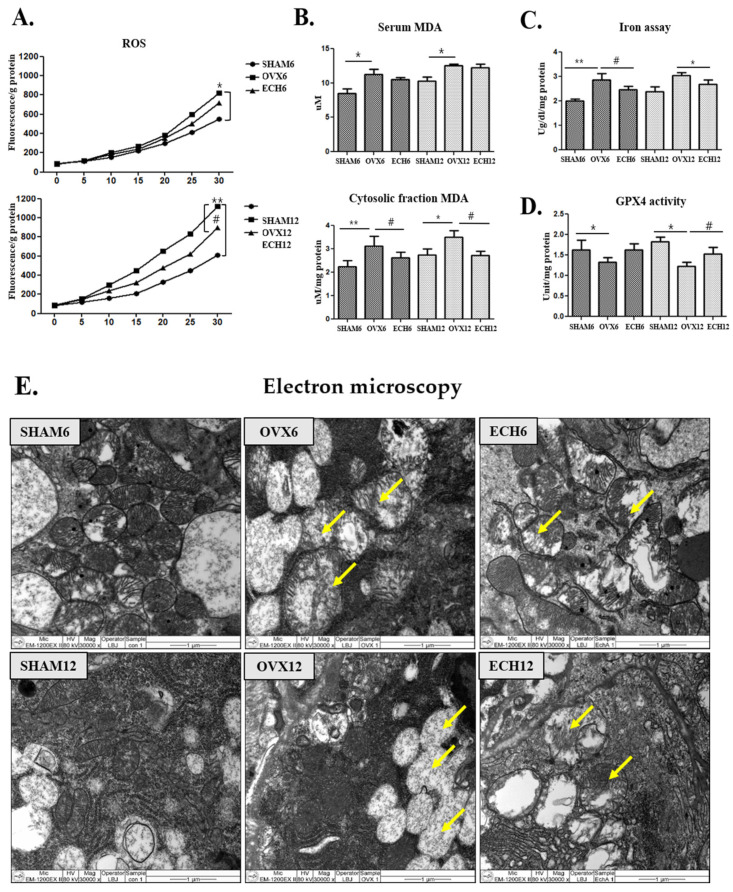
Effect of Ech A on ferroptosis response. The submandibular gland ferroptosis response was elevated in SHAM groups but diminished following Ech A treatment. (**A**) ROS; reactive oxygen species generation. ROS generation was higher in the OVX groups than in the SHAM groups (OVX-6 vs. SHAM-6; *p* < 0.05 and OVX-12 vs. SHAM-12; *p* < 0.01) but reduced significantly in the ECH group (ECH-12 vs. SHAM-12; *p* < 0.05). (**B**) Malondialdehyde (MDA) concentration in the serum and submandibular gland tissue. Submandibular gland cytosolic fraction MDA level significantly decreased in the Ech A treatment group (ECH-6 and 12 vs. SHAM-6 and 12, respectively; *p* < 0.05 compared to SHAM-6 and 12). (**C**) Iron deposition was significantly increased in the OVX groups (OVX-6 vs. SHAM-6; *p* < 0.01 and OVX-12 vs. SHAM-12; *p* < 0.05). In the ECH-6 group, iron deposition was significantly reduced compared to OVX-6. (**D**) Glutathione peroxidase 4 (GPX4) activity was suppressed in both OVX groups (OVX-6 and 12 vs. SHAM-6 and 12, respectively; *p* < 0.05) but significantly up-regulated with Ech A treatment (ECH-12 vs. SHAM-12; *p* < 0.05). (**E**) Electron microscopy of submandibular glands revealed swollen mitochondria and destruction of mitochondrial crests were observed in the OVX groups, while the Ech A treatment improved mitochondrial ultrastructure, resulting in the reappearance of an electron-dense matrix and improve cristae. *n* = 8, each group. Error bar; S.D. One-way ANOVA; * *p* < 0.05, ** *p* < 0.01; vs. SHAM, # *p* < 0.05; vs. OVX.

The effects of Ech A on ovariectomy-induced cytosolic oxidative damages were explored. ROS generation was analyzed using the dichlorofluorescein diacetate (DCF-DA) assay. Ech A treatment significantly reduced the elevated ROS levels in the OVX groups. These results confirmed that Ech A exerted an ROS scavenging effect (Figure 4A). Elevated levels of malondialdehyde (MDA), an indicator of lipid peroxidation, were detected in the serum and cytosolic fractions of the salivary gland tissues of the OVX groups (Figure 4B). The ECH group, however, showed a marked reduction in submandibular gland cytosolic fraction MDA levels than the OVX groups (Figure 4B). Iron deposition was significantly higher in the OVX groups than in the SHAM groups, significantly lower in the ECH-6 group than the OVX-6 group, with no difference between ECH-12 and OVX-12 groups. These findings indicated that Ech A inhibited ovariectomy-induced iron deposition in the submandibular gland tissue (Figure 4C). GPX4, a potent biological antioxidant, catalyzes the reduction of hydrogen and lipid peroxides using reduced glutathione to protect cells against oxidative stress. Ech A treatment significantly upregulated GPX4 activity in the ECH-12 group compared with the OVX-12 group (Figure 4D). Mitochondria are polymorphic, with a condensed matrix and blurred internal structures. The mitochondria are the primary targets of the damaging oxidative stress effects. Submandibular gland cells from the OVX groups showed mitochondrial swelling and crest degeneration (Figure 4E). However, Ech A treatment improved the mitochondrial ultrastructural features.

### 2.5. Ech A Reduced Inflammation and Fibrosis

**Figure 5:**
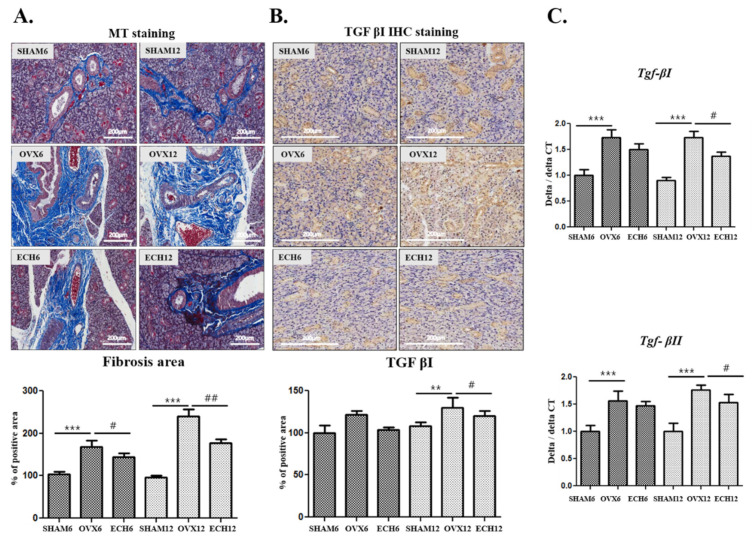
Effect of Ech A on inflammation and fibrosis. Submandibular gland inflammation and fibrosis were increased in SHAM groups but decreased in the ECH groups. (**A**) Fibrotic area detected by Masson’s trichrome staining. Peri-striated ductal and perivascular fibrosis also increased in the OVX groups (OVX-6 and 12 vs. SHAM-6 and 12, respectively; *p* < 0.05) but decreased with Ech A treatment (ECH-6 vs. OVX-6; *p* < 0.05 and ECH-12 vs. OVX-12; *p* < 0.01). (**B**) TGF-β1-positive stained regions were significantly higher in the OVX-12 group (*p* < 0.01 compared to SHAM-12), but significantly reduced in the ECH-12 group (*p* < 0.05 compared to OVX-12). (**C**) Tgf-β1 and Tgf-βII mRNA expression was significantly upregulated in the OVX groups (OVX-6 and 12 vs. SHAM-6 and 12, respectively; *p* < 0.05), while they were downregulated in the ECH-12 group (*p* < 0.05 compared to OVX-12). *n* = 8, each group. One-way ANOVA; ** *p* < 0.01, *** *p* < 0.001; vs. SHAM, # *p* < 0.05, ## *p* < 0.01; vs. OVX.

Submandibular gland fibrosis was confirmed using Masson’s trichrome staining. As depicted in Figure 5A, fibrosis was elevated in the OVX groups but decreased with Ech A treatment. The immunohistochemical analysis also confirmed OVX-associated fibrosis. Transforming growth factor β (TGF β) is a critical fibrogenesis mediator. TGF-β is upregulated and activated in the fibrotic tissues [30]. Specifically, TGF-β1 is a major fibrotic factor that induces fibrosis. Figure 5B reveals that the OVX-12 group had higher TGF-β1-positive stained regions than the ECH-12 group. In addition, the mRNA expression of Tgf-βI and Tgf-βII was significantly upregulated in the OVX groups but downregulated in the ECH-12 groups (Figure 5C).

### 2.6. Ech A Improved Submandibular Gland Functions

**Figure 6:**
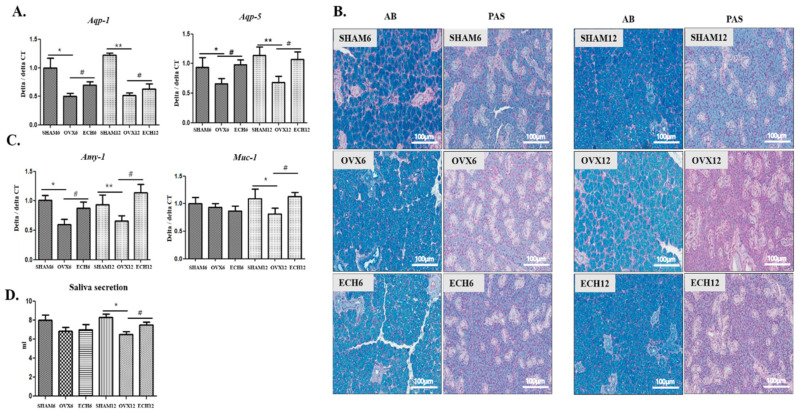
Effect of Ech A on salivary gland function. Submandibular salivary gland function decreased in the SHAM groups and improved in the Ech A treatment groups. (**A**) Aqp-1 and Apq-5 mRNA expression significantly diminished in the OVX groups (OVX-6 vs. SHAM-6; *p* < 0.05 and OVX-12 vs. SHAM-12; *p* < 0.15), while they were elevated in the ECH groups (ECH-6 and 12 vs. OVX-6 and 12; *p* < 0.05). (**B**) Alcian blue (AB) and Alcian blue-periodic acid-Schiff (AB-PAS) staining was weaker in the OVX groups, and stronger in the Ech A treatment groups. (**C**) Quantitative polymerase chain reaction (qPCR) analysis of Amy-1; amylase and Muc-1; mucin, these enzymes decreased in the OVX groups, but increased in the ECH groups. (**D**) Saliva secretion decreased in the OVX-12 group (*p* < 0.05) whereas it increased with Ech A treatment (*p* < 0.05). n = 8, each group. Error bar; S.D. One-way ANOVA; * *p* < 0.05 ** *p* < 0.01; vs. SHAM, # *p* < 0.05 vs. OVX.

The mRNA expression of aquaporin (Aqp)-1 and 5 was investigated to evaluate whether Ech A improved the submandibular gland functions. As shown in Figure 6A, the mRNA expression of Aqp-1 and Aqp-5 was significantly reduced in the OVX groups whereas it was elevated in the ECH groups. Moreover, the acidic and neutral mucin can be detected using Alcian blue (AB) and periodic acid-Schiff (PAS) staining, respectively. As shown in Figure 6B, the OVX groups exhibited light staining, indicating that less acidic and neutral mucins accumulated in the OVX groups. However, AB and PAS staining intensity were higher, respectively, in the ECH-12 group and both ECH-6 and 12 groups, indicating that more acidic and neutral mucins were increased with Ech A treatment. Both amylase (Amy-1) and mucin (Muc-1) mRNA expression were also significantly decreased in the OVX groups while elevated in the ECH groups (Figure 6C). Finally, as shown in Figure 6E, saliva secretion decreased in the OVX-12 group but significantly increased with Ech A treatment (Figure 6D).

## 3. Discussion

Post-menopausal xerostomia is a severe symptom impairing the quality of life of women. Although menopause-related xerostomia has been documented, the precise mechanism remains ambiguous. Ferroptosis induction in the submandibular glands of ovariectomized rats is a critical mechanism relating to salivary dysfunctions [11]. Therefore, ferroptosis inhibition is a proposed mechanism for improving salivary gland functions. However, the mechanism of ferroptosis-induced xerostomia has not yet been explored. Moreover, there is currently no gold-standard treatment for xerostomia.

Estrogen replacement therapy effectively alleviates menopausal symptoms such as xerostomia and hot flashes [4,31]. While the efficacy of hormone replacement therapy is undeniable, there are safety concerns. Tissue engineering and regenerative medicine are being investigated as prospective therapeutic modalities for salivary gland function recovery after menopause. Previous research has shown that mesenchymal stem cells extracted from bone marrow or the umbilical cord can aid in the healing of ovariectomy-induced damage to the parotid glands [32,33]. Moreover, tonsil mesenchymal stem cell-derived extracellular vesicles protect against the ovariectomy-induced deterioration of submandibular gland functions [34]. However, treatment using these stem cells or exosomes is presumed to act via anti-inflammatory and anti-fibrotic mechanisms without affecting the underlying mechanism of post-menopausal salivary gland dysfunction. Consequently, a method that is based on the mechanism of salivary gland dysfunction after menopause could be appropriate and effective.

A reduction in salivary gland function after menopause has been linked to ferroptosis [9]. Ferroptosis is programmed cell death caused by iron-dependent ROS and lipid accumulation, thereby promoting lipid peroxidation [35,36]. Excessive lipid peroxidation plays a significant role in promoting ferroptosis. The primary defense strategy in ferroptosis is the synthesis of endogenous antioxidants such as glutathione (GSH), coenzyme Q10 (CoQ10), and tetrahydrobiopterin (BH4) [37,38]. Salivary gland function may be restored after menopause if ferroptosis is inhibited; however, this has not yet been reported. Antioxidants might be effective for post-menopausal dry mouth by inhibiting ferroptosis. In this study, we observed that the antioxidant Ech A decreased ferroptosis by decreasing ROS generation, inhibiting lipid peroxidation, increasing GPX4, and reducing iron deposition. Furthermore, Ech A suppressed Srebp-1c, Acc, Fasn, and Cd36 in salivary gland tissue, indicating its effectiveness in reducing fat production. Therefore, Ech A is proposed to protect against salivary gland malfunction after menopause by lowering inflammation and fibrosis via its effects on ferroptosis and fat production.

Some studies propose that antioxidative agents may improve salivary gland function after menopause. Xu et al. reported that dietary nitrate improved salivary gland function in OVX rats by suppressing apoptosis and upregulating Cu-Zn SOD expression [32]. Consequently, this group suggested that a nitrate-rich diet could minimize the incidence of hyposalivation in post-menopausal women. Da et al. highlighted that Cimicifuga racemosa methanol extracts with antioxidant properties promote salivary gland recovery. These extracts reduce cleaved caspase-3 expression and increase Cu-Zn SOD activity in post-menopausal salivary glands [39]. Additionally, Kim et al. revealed that oligonol, an antioxidant, aids in the restoration of post-menopausal salivary gland functions by inhibiting lipogenesis and reducing lipid peroxidation [40]. Oligonol also induced biogenesis and dynamic modifications in mitochondria, which were involved in restoring salivary gland function.

Consistent with an earlier study, the present investigation suggests that ferroptosis may be implicated in menopause-induced submandibular gland dysfunction [9]. Researchers have confirmed that oligonol, another antioxidant, had similar effects to Ech A in lowering lipogenesis-related gene expression in menopausal salivary glands. However, this is the first study to establish that Ech A inhibits ferroptosis, preventing salivary gland dysfunction and fibrosis after menopause. This is accomplished by lowering lipid peroxidation, increasing GPX4 activity, and decreasing iron deposition. Ech A has been shown to reduce salivary gland dysfunction in a menopausal dry mouth model by decreasing lipogenesis and ferroptosis; however, the precise cellular and molecular mechanism are yet to be characterized. This is because this investigation was conducted in vivo. There is currently no in vitro model that can accurately mimic the post-menopausal state.

## 4. Materials and Methods

### 4.1. Echinochrome A

We used Histochrome^®^ (Reg. No. P N002363/01, expiration data; 0111115, 11/18). Although echinochrome A is water-insoluble, the Histochrome^®^ drug, a water-soluble sodium salt of echinochrome A, is used for medical applications and is manufactured in ampoules under inert conditions. Histochrome^®^ was generated by combining echinochrome A (1 g) with sodium carbonate (0.4 g) in a water solution heated in inert gas until the CO_2_ was completely removed and sealed in ampoules in inert gas. Histochrome^®^ has applications in the domains of ophthalmology and cardiology in Russia. Histochrome^®^ was kindly provided by the G.B. Elyakov Pacific Institute of Bioorganic Chemistry, Far-Eastern Branch of the Russian Academy of Science.

### 4.2. Experimental Design

A total of forty-eight eight-week-old female Sprague-Dawley rats were used in this study (Central Lab, Seoul, Republic of Korea). Each group was weight-matched at the beginning of the study. The animals were randomly assigned to one of six groups after a week of acclimation: sham-operated rats (SHAM-6 and -12), ovariectomized rats (OVX-6 and -12), and Ech A-treated ovariectomized rats (ECH-6 and -12). These groups comprised of 6- and 12-weeks post-sham-operated, ovariectomized, and Ech A-treated ovariectomized rats, respectively. All the rats were maintained in a pathogen-free environment on a 12 h light/dark cycle and provided rat chow with water ad libitum. For OVX surgery, the rats were anesthetized using isoflurane inhalation (3% dissolved in oxygen), and an incision was made at the midline of the abdomen with the bilateral ovaries being revealed. In the OVX group, the ovaries were ligated and severed bilaterally before closing the abdominal cavity. Following ovarian excision, the rats were injected intraperitoneally with 10 mg/kg Ech A three times a week for 6- (ECH-6) and 12-weeks (ECH-12). The body weight and food intake were measured once a week. We supplied enough food of the same weight once a week and measured the weight of the remaining food after one week to calculate the weight of the food that was eaten for one week. The study was approved by the Institutional Animal Care and Ethics Committee of Pusan National University Hospital (No. PNUH-2020-172).

### 4.3. Serum Estradiol Analysis

Serum estradiol (E2) concentrations were measured using rat-specific estradiol enzyme-linked immunosorbent (ELISA) assay plates that were coated with a biotin-conjugated binding protein kit purchased from Abcam Incorporation (285285, Cambridge, MA, USA). A cardiac puncture was performed, and the blood was centrifuged at 3000 rpm for 30 min. The plasma was immediately frozen in liquid nitrogen and stored at −80 °C.

### 4.4. Tissue Preparations

Frozen submandibular gland tissue was homogenized in hypotonic lysis buffer [buffer A: 10 mM KCl, 2 mM MgCl2, 1 mM dithiothreitol (DTT), 0.1 mM ethylenediaminetetraacetic acid (EDTA), 0.1 mM PMSF, 1 mM pepstatin, 2 mM leupeptin, 20 mM β-glycerophosphate, 20 mM NaF, 2 mM Na3VO4, 10 mM HEPES, pH 7.4] using a tissue homogenizer (Homogenizing Stirrer, Daihan Science, HS-30E, Korea) for 20 s. The amount of buffer was determined by counting triplicate of the tissue weight. After cooling the homogenates on ice for 15 min, 125 μL of 10% Nonidet P-40 (NP-40) solution was added, mixed for 15 s, and centrifuged at 14,000× *g* for 2 min. The supernatants were subsequently used as the cytosolic fraction after being stored at −80 °C. The protein concentration was determined using the bicinchoninic acid (BCA) assay (23225, Thermofisher [41].

### 4.5. Staining and Immunohistochemistry Analysis

Submandibular glands from each rat were isolated and fixed overnight in 4% formalin. We used an automatic tissue processor for paraffin embedding (Leica, TP1020, semi-enclosed benchtop tissue processor, Wetzlar, Germany) and dispensing (Leica EG1150H, heated paraffin embedding module). De-paraffinized sections (8 µm thick) were placed on glass slides and stained with hematoxylin-eosin, Masson’s trichrome, Nile red, Alcian blue, and Alcian blue-periodic acid-Schiff. For immunohistochemistry, de-paraffinized sections were incubated for 24 h at 4 °C with anti- TGF βI (sc130348, 200 ug/mL) primary antibodies (Santa Cruz Biotechnology, Dallas, TA, USA). After the primary antibody was removed by rinsing, the sections were incubated with goat-anti-rabbit secondary antibodies (1:1000) (ENZO Biochem, New York, NY, USA) for 1 h at room temperature) and double-stained with DAB (3,3-diaminobenzidine). Incubation in phosphate-buffered saline that was supplemented with 1% bovine serum albumin instead of the primary antibody served as a negative control. We selected specific sections of submandibular gland tissue for representative images captured at 200× using a light microscope (Leica DM4000/600M, Versatile upright microscope for materials analysis). Morphometric determination was performed using 40× images of whole submandibular gland tissues using the morphometric method with an image analysis system software (Leica Basic LAS V3.8 software, Wetzlar, Germany).

### 4.6. Quantitation of Redox Status and Ferroptosis Response

ROS generation was measured using a specific fluorescent probe. The homogenates were treated with a 250 μL final volume of 25 μM of 2′, 7′-DCF-DA. The fluorescence intensity was monitored every 5 min for 30 min on a fluorescence plate reader at excitation and emission wavelengths of 485 and 530 nm, respectively. The MDA concentration was determined using the Bioxytech LPO-586 assay kit (OXIS Health Products, Foster, CA, USA) with an absorbance plate reader at 580 nm. Serum Fe concentrations were measured using a rat-specific colorimetric iron assay kit (Biovision Incorporation, Spring Valley, CA, USA). Ferric carrier proteins dissociate ferric into solution in the presence of an acid buffer. After reduction to the ferrous state (Fe²^+^), iron reacts with Ferene S to produce a stable colored complex with an absorbance at 593 nm. GPX4 activity was analyzed using a glutathione peroxidase 4 assay kit (Abcam, Cambridge, MA, USA). The kit uses a chromogenic reagent that reacts with the lipid peroxidation product, malondialdehyde, yielding a stable chromophore with maximum absorbance at 586 nm.

### 4.7. Electron Microscopy

The material was fixed with 2.5% glutaraldehyde (4 °C, phosphate buffer, pH 7.4) and 1% osmium tetroxide in the same buffer. The material was dehydrated with a series of graded ethyl alcohol solutions and embedded in epoxy resin (Epon 812 mixture). Thick sections (1 µm) were stained with 1% toluidine blue for light microscopy. Thin sections (50~60 nm) were prepared using an ultramicrotome (EM UC7, Leica, Wetzlar, Germany), which were double stained with uranyl acetate and lead citrate. Thin sections were examined using a transmission electron microscope (JEM-1200EXⅡ, JEOL, Tokyo, Japan).

### 4.8. Quantitative Real-Time PCR

Submandibular gland RNA was extracted using TRIzol (Life Technologies, Rockville, MD, USA). A reverse transcription kit (Applied Biosystems, Foster City, CA, USA) was used to perform reverse transcription, according to the manufacturer’s protocol. The RNA concentration was determined using a NanoDrop^®^ ND-1000 spectrophotometer. The ratio of absorbance at 260 nm/280 nm was used to determine RNA purity. qPCR was performed according to the SYBR ^®^ Green PCR protocol (Applied Biosystems, Foster City, CA, USA). The reaction conditions were: 10 min at 95 °C (one cycle), 10 s at 95 °C, and 30 s at 60 °C (40 cycles). Gene-specific PCR products were continuously measured using an ABI PRISM 7900 HT Sequence Detection System (PE Applied Biosystems, Waltham, CT, USA). The primer sequences are presented in Table 1. The delta CT/target gene delta CT ratio was calculated via normalization by comparing the cycle threshold differences (delta CT) to the Gapdh expression level.

### 4.9. Saliva Secretion

The secretion of saliva was induced by subcutaneous injection of phyllocarpine (2 mg/kg weight). Pilocarpine injection was performed under deep anesthesia with isofluorane (3% dissolved in oxygen). After injection, the mouth was wiped with a cotton swab and filled with cotton balls. In order to prevent saliva loss, a 50 mL tube was placed over the head of the rat and fixed in the anesthesia box, and the head of the rat was installed so that it descended at an oblique angle. In order to avoid errors, this treatment was performed equally for all mice within 5 min, and saliva was collected up to 30 min after pilocarpine injection in the same anesthesia box. After 30 min, the total weight of secreted saliva and (including the weight of cotton balls before and after collection) was evaluated.

### 4.10. Statistical Analysis

Unless otherwise specified, all quantitative results were presented as the mean and standard error of the mean from at least three independent experiments. The statistical significance between the groups was established using a one-way analysis of variance (ANOVA) with *p* < 0.05 considered statistically significant.

## 5. Conclusions

Ech A, as an antioxidant, protects against post-menopausal salivary gland dysfunction by inhibiting lipogenesis and ferroptosis. These findings suggest that Ech A is effective for the treatment of menopausal dry mouth.

## Figures and Tables

**Table 1:** Primer sequences of the different target genes analyzed using PCR.

Gene	Direction	Sequence
*Erβ*	Forward	GAAGCTGAACCACCCAATGT
	Reverse	CAGTCCCACCATTAGCACCT
*Srebp-1c*	Forward	CTGTCGTCTACCATAAGCTGCAC
	Reverse	ATAGCATCTCCTGCACACTCAGC
*Acc*	Forward	AACATCCCGCACCTTCTTCTAC
	Reverse	CTTCCACAAACCAGCGTCTC
*Fasn*	Forward	TCCCAGGTCTTGCCGTGC
	Reverse	GCGGATGCCTAGGATGTGTGC
*Cd36*	Forward	GATGACGTGGCAAAGAACAG
	Reverse	TCCTCGGGGTCCTGAGTTAT
*Tgf-βI*	Forward	GACGTTCGCCATAACCAAGT
	Reverse	CTGCAGGTTCTCAATGCAAA
*Tgf-βII*	Forward	CCAATCACGCAATAGTTCTGG
	Reverse	CGCTGTATCGTATGGCGAT
*Aqp-1*	Forward	CCTGCTGGCCATTGACTACA
	Reverse	TGGTTTGAGAAGTTGCGGGT
*Aqp-5*	Forward	CATGAACCCAGCCCGATCTT
	Reverse	AGAAGACCCAGTGAGAGGGG
*Amy-1*	Forward	GCAACCAAGTAGCTTTTGGCA
	Reverse	TGCCATCGACTTTGTCTCCAG
*Muc-1*	Forward	GAGTGAATATCCTACGACCAC
	Reverse	TTCACCAGGCTAACGTGGTGAC
*Gapdh*	Forward	ACCCCCAATGTATCCGTTGT
	Reverse	TACTCCTTGGAGGCCATGTA

## Data Availability

The data that support the findings of this study are available on request from the first and corresponding authors.
